# Supplementary material for: Yoga and Multiple Sclerosis: Maintaining engagement in physical activity
Source: PLoS One. 2023 Jul 19;18(7):e0288319. doi: 10.1371/journal.pone.0288319 (PMC10355448; doi:10.1371/journal.pone.0288319)
Supplement: Attachment — Submitted filename: Response to Reviewers Comments.docx [file pone.0288319.s001.docx]

Dear reviewers,

Very many thanks for your helpful comments on the manuscript. We have addressed all areas and feel this is a much-improved paper. The responses to each reviewer point are in the table below.

Information about informed consent has been added to lines 129-133. The data availability statement is located on lines 548-552.

Kind regards

Dr Jenni Naisby

| **Comment** | **Response** |
| --- | --- |
| A strong introduction, discussion and conclusion parts needed with up to date literature review and (2020 and above) and practical suggestions for the future researchers. | More recent literature has been added to the introduction (Legault *et al.* 2021) line 109 and conclusion lines 423-425 (Fasczewski *et al.* 2022) and lines 467-469. (Lysigirskaia *et al.* 2023).  Practical suggestions have been added to the conclusion line 533. |
| Reliability and validity of the data should be reported. | A section on Trustworthiness has been added to the discussion lines 505-508. |
| Each individual characteristics should be defined in detail such as gender, race, education and culture | The participant information we have is detailed line 126-130. We have gender, but did not collect race, education and culture. |
| I recommend the authors to give more details on the protocol and Yoga sessions conducted. The frequency, the yoga poses adapted or not adapted to the needs of the participants. | The class contents have been added to lines 137-143 and details of how these can be adapted. |
| Since, YOGA can be individualized as movement intervention, I would like to see the how the increased awareness and knowledge on MS could contribute to the further individualization to YOGA sessions. | Individualisation of yoga and MS has been added to lines 432-439. |
| It is possible to discuss the role of the breathing exercises more on the positive feelings and wellbeing. Moreover, if the combination of Yoga poses and breathing augmented the effect, it would be good to have some science based ideas about this link. | Further detail has been added regarding the role of breathing, yoga, wellbeing, and fatigue on lines 441-450. |
| Please describe if there is any specific information on the degree or level of participant's MS even the average age. | The participant information we have is detailed on lines 126-130. We did not collect information on level of MS or age. |
| The words "sense of working hard" and "sense of achievement" could be mentioned in the results and discussion to strengthen the link between the conclusion and the results. | Sense of working hard and achievement is included on lines 191-192. Sense of working hard is included in line 467 and 532. Sense of achievement is mentioned within the discussion on line 467. |
